# Effect of Immunoglobin-Like Transcript 7 Cross-Linking on Plasmacytoid Dendritic Cells Differentiation into Antigen-Presenting Cells

**DOI:** 10.1371/journal.pone.0089414

**Published:** 2014-02-19

**Authors:** Barbara Tavano, Adriano Boasso

**Affiliations:** 1 Immunology Section, Chelsea and Westminster Hospital, Imperial College, London, United Kingdom; Rush University, United States of America

## Abstract

Plasmacytoid dendritic cells (pDC) are innate immunity effector cells which play a critical role in the transition from innate to adaptive immune response. Circulating blood pDC present an immature phenotype and can differentiate into either antigen-presenting cells (APC) or type I interferon (IFN-I)-producing cells (IPC). The immunoglobulin-like transcript (ILT)7 is a surface receptor expressed by immature pDC, and ILT7 cross-linking (XL-ILT7) inhibits IFN-I production by pDC in response to toll-like receptor (TLR)7 and 9 stimulation. We used peripheral blood mononuclear cells (PBMC) from healthy donors to test the effect of XL-ILT7 on 1) TLR7/9-mediated regulation of gut mucosal (α4β7 integrin) and lymph node (CCR7) migration markers; and 2) the maturation of pDC into APC. We found that XL-ILT7 mitigated the upregulation of CCR7 and enhanced that of β7 on TLR7/9-stimulated pDC. TLR7/9 stimulation induced upregulation of CD40, CD80 and CD86. CD40 expression was partially reduced by XL-ILT7, whereas CD86 was further enhanced. Plasmacytoid DC stimulated with TLR9 ligand in presence of XL-ILT7 retained the ability to induce T cell proliferation and activation in response to staphylococcal enterotoxin B (SEB) in pDC-T cell co-cultures. Our results suggest that XL-ILT7 favours the differentiation of immature pDC into APC rather than IPC.

## Introduction

Blood plasmacytoid dendritic cells (pDC) are immature lymphoid-derived DC which have the ability to capture and process antigens. Upon antigen capture, pDC migrate to the lymph nodes and mature into professional antigen presenting cell (APC), which present peptides to T cells in association with MHC molecules [1]. In particular, the expression of specific pattern recognition receptors (PRR), especially Toll-like receptors (TLR), confer them the ability to respond to viral pathogens. The expression of endosomal TLR7 and TLR9 renders pDC responsive to single-stranded RNA and unmethylated CpG-rich DNA respectively, which are distinctive of most viral genomes [2], [3]. Following exposure to viruses or nucleic acid, pDC produce large quantities of type I interferon (IFN-I) [2] which create a cellular environment hostile for viral activity by: 1) limiting the uptake of nutrients from the extracellular compartment; 2) promoting RNA degradation; and 3) inducing anti-proliferative or pro-apoptotic mechanisms in different cell types including T cells [4]. Additionally, upon TLR stimulation, pDC upregulate indoleamine-2,3-dioxygenase (IDO), an enzyme involved in the catabolism of the essential amino acid tryptophan [5], [6]. Thus, activated pDC also exert a negative regulatory activity on T cells which is essential for the maintenance of immunologic tolerance [6]–[10]. When persistently activated, for example during chronic viral infections or cancer, pDC contribute to the detriment of the immune system in both human [6], [11]–[13] and animals models [14], [15].

The Immunoglobulin-like transcript 7 (ILT7), also known as CD85g or leukocyte immunoglobulin-like receptor subfamily A member 4 (LILRA4), is a protein of 499 amino acids which presents the typical morphology of ILT stimulatory receptors and is exclusively expressed by human pDC [16]–[18]. ILT7 cross-linking inhibits TLR7/9-mediated IFN-I production by pDC [18], [19]. Although the IFN-I-inducible molecule bone marrow stromal antigen 2 (BST2) was identified as a ligand inducing ILT7 cross-linking [20], we have recently shown that BST2 may not play a biologically relevant role in primary human circulating pDC [21]. Our data indicated that ILT7 is rapidly downregulated *in vitro* during spontaneous pDC differentiation, defined by an increase of the pDC morphological complexity and CCR7 expression [21]. In contrast, CD83 upregulation, a marker of full pDC activation and maturation, occurred only following TLR7/9 stimulation [21]. Thus, we hypothesized that ILT7 cross-linking may be involved in the homeostatic modulation of immature circulating pDC rather than provide a negative feedback for activated pDC [21].

In the present study, we built upon our previous work and tested the effect of ILT-7 cross-linking (XL-ILT7) on the ability of TLR7/9-stimulated pDC to express gut and lymph node migration markers and mature into APC. We found that XL-ILT7 favored the upregulation of the gut-migration integrin α4β7 and mitigated the upreulation of CCR7, which mediates migration to lymphoid tissues. XL-ILT7 had mild effect on the expression of CD40 and CD86, but did not interfere with the overall maturation of pDC in APC and with their ability to activate T cells.

## Materials and Methods

### Ethics Statement

Leukoreduction system chambers (LRSC) from healthy blood-bank donors were purchased as non clinical blood components from the North London Blood Transfusion Service (UK). The blood donor consent procedure includes provision for such materials to be used to benefit patients indirectly, including ethically approved research (full details available at http://hospital.blood.co.uk). The study was approved by the Riverside Research Ethics Committee.

### Blood Samples and Leukocyte Isolation

Peripheral blood mononuclear cells (PBMC) were isolated by density gradient centrifugation using Histopaque-1077 (Sigma-Aldrich, Poole, U.K.) and cultured at 2×10^6^ cells/ml in RPMI 1640 (PAA Laboratories, Pasching, Austria), 10% FBS (Sigma-Aldrich), and 1% Pen-Strep-Glut (Sigma-Aldrich).

### Plasmacytoid DC and Autologous T Cell Isolation

PBMC were resuspended in PBS containing 2% FBS and further separated into high and low density fractions by 50% Percoll gradient centrifugation. The interface layer, containing the monocyte/DC enriched fraction, was harvested and washed twice; pDC were then magnetically isolated using a CD304 (BDCA-4/Neuropilin-1) MicroBead kit (Miltenyi Biotec, Germany) in accordance with the manufacturer’s protocol. The Percoll pellet, enriched for lymphocytes, was washed twice and used for the negative selection of autologus T cell using the Pan T cell Isolation Kit II (Miltenyi Biotec, Germany) in accordance with the manufacturer’s protocol. Isolation of pDC and T cells using this methods yielded purities of at least 93% and 92%, respectively. Plasmacytoid DC and T cells were then co-cultured at 1∶10 ratio in RPMI 1640 (PAA Laboratories), 10% FBS (Sigma-Aldrich), and 1% Pen-Strep-Glut (Sigma-Aldrich) for 72 hours before analysis of T cell activation and proliferation.

### TLR Ligands, ILT7 Cross-linking and SEB Stimulation

PBMC and T cell/pDC co-cultures were stimulated or not with TLR agonists and cross-linking (XL)-ILT7 ab depending on the experimental setting, as described in the Results section. TLR9 ligand (TLR9L) CpG ODN type A (Invivogen, San Diego, CA) was used at 0.75 µM final concentration. TLR7 ligand (TLR7L) R848 (Imiquimod; Invivogen) was used at 5 µg/ml final concentration. XL-ILT7-specific Ab 17G10.2 (eBioscence, Hatfield, U.K.) was used at 10 µg/ml final concentration in plate bound form and cells were pre-incubated for 30 min before stimulation with TLR7/9L. Culture plates were coated by overnight incubation at 4°C with 10 µg/ml 17G10.2 Ab in 100 µl (96-well plates) or 200 µl (48-well plates) of PBS. The PBS was discarded after overnight incubation. Plate bounded mouse IgG1 isotype was used as control.

The superantigen staphylococcal enterotoxin B (SEB) (Sigma-Aldrich) was used at 5 µg/ml final concentration in T cell/pDC co-culture.

### Flow Cytometry

Cell were incubated for 20 min at room temperature with different combinations of the following anti-human Abs: CD83 Phycoerythrin (PE) clone HB15e, CD8 allophycocyanin (APC) clone SK1, CD80 Fluorescein isothiocyanante (FITC) clone 2D10.4, α4 PE clone 9F10, β7 FITC clone FIB504, CD4 PE clone RPA-T4, CD38 FITC clone HB7 (all purchased from eBioscence); CD123 PE-Cy7 clone 6H6, CD86 Peridinin Chlorophyll Protein (PrCP Cy5.5) clone IT2.2, CD40 Pacific Blue clone 5C3, CD69 PrCP Cy5.5 clone FN50 (purchased from BioLegend London, U.K.); CD14 allophycocyanin -Hilite.7 (APC H7) clone 6MPφ9, CCR7 PrCP Cy5.5 clone 150503 (purchased from BD Biosciences); BDCA2 (CD303) APC clone AC144 (purchased from Miltenyi Biotec). Cells were washed with staining buffer (BD Biosciences) and fixed with BD cytofix buffer (BD Biosciences). FACS analysis was performed on a LSR-II flow cytometer using FACSDiva software (BD Biosciences). FlowJo software (Tree Star, Ashland, OR) was used for data analysis. Fluorescence-minus-one controls were used to establish positivity thresholds.

### Proliferation Assay

T cells proliferation was evaluated using a flow cytometry-based intracellular dye dilution proliferation assay, based on the Violet Proliferation Dye 450 (VPD450; BD Bioscience). VPD450 staining was carried out according the manufacture’s protocol.

### Statistical Analysis

Statistical analyses were performed using SPSS 20.0 software (SPSS, Chicago, IL). Pairwise comparisons (control versus TLR7/9-stimulated cells; and TLR7/9-stimulated in presence versus absence of XL-ILT7) were performed using nonparametric Wilcoxon sign rank test. *p* values <0.05 were considered statistically significant.

## Results

### ILT7 Cross-linking did not Affect pDC Survival and Inhibited TLR9-induced IFN-α Production

**Figure 1:**
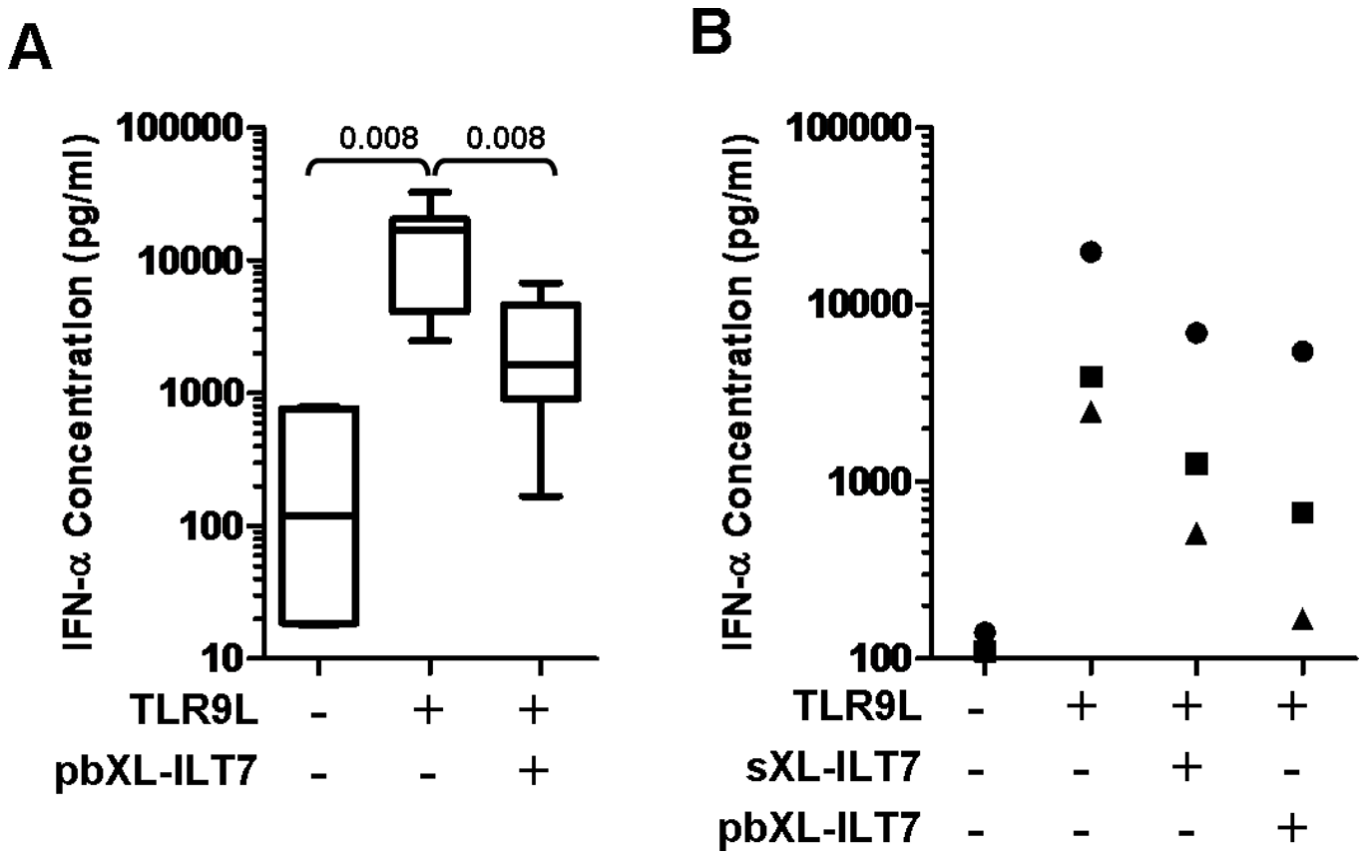
Effect of ILT7 cross-linking on TLR9-induced IFN-α production. PBMC from healthy donors were cultured overnight with TLR9L in the absence (isotype control) or presence of soluble or plate bound XL-ILT7 (sXL-ILT7 and pbXL-ILT7, respectively). Panel (**A**) shows box and whiskers plots summarizing IFN-α levels in supernatants from N = 9 independent experiments performed using pbXL-ILT7; horizontal lines within boxes indicate median values, boxes indicate 25^th^ and 75^th^ percentiles, and vertical lines extend to 10^th^ and 90^th^ percentile; numbers above brackets indicate P values for pair wise comparisons (Wilcoxon sign rank test). Panel (**B**) shows a comparison between sXL-IL7 and pbXL-ILT7 on N = 3 independent experiements; each symbol represents results from one individual donor.

Freshly isolated PBMC were cultured overnight in presence or absence of TLR7/9L and pre-incubated or not with plate bound XL-ILT7 Ab. As previously reported, overnight TLR9L stimulation of PBMC induced significant IFN-α production, which was inhibited using either plate bound or soluble XL-ILT7 Ab (Fig. 1A and 1B). Plate bound XL-ILT7 proved more potent than soluble XL-ILT7 Ab in downregulating TLR9-mediated IFN-α production (83%±12% versus 71%±9% inhibition; Fig. 1B). Plate bound XL-ILT7 Ab has been used for all experiments described, and will be referred to simply as XL-ILT7 from this point on.

**Figure 2:**
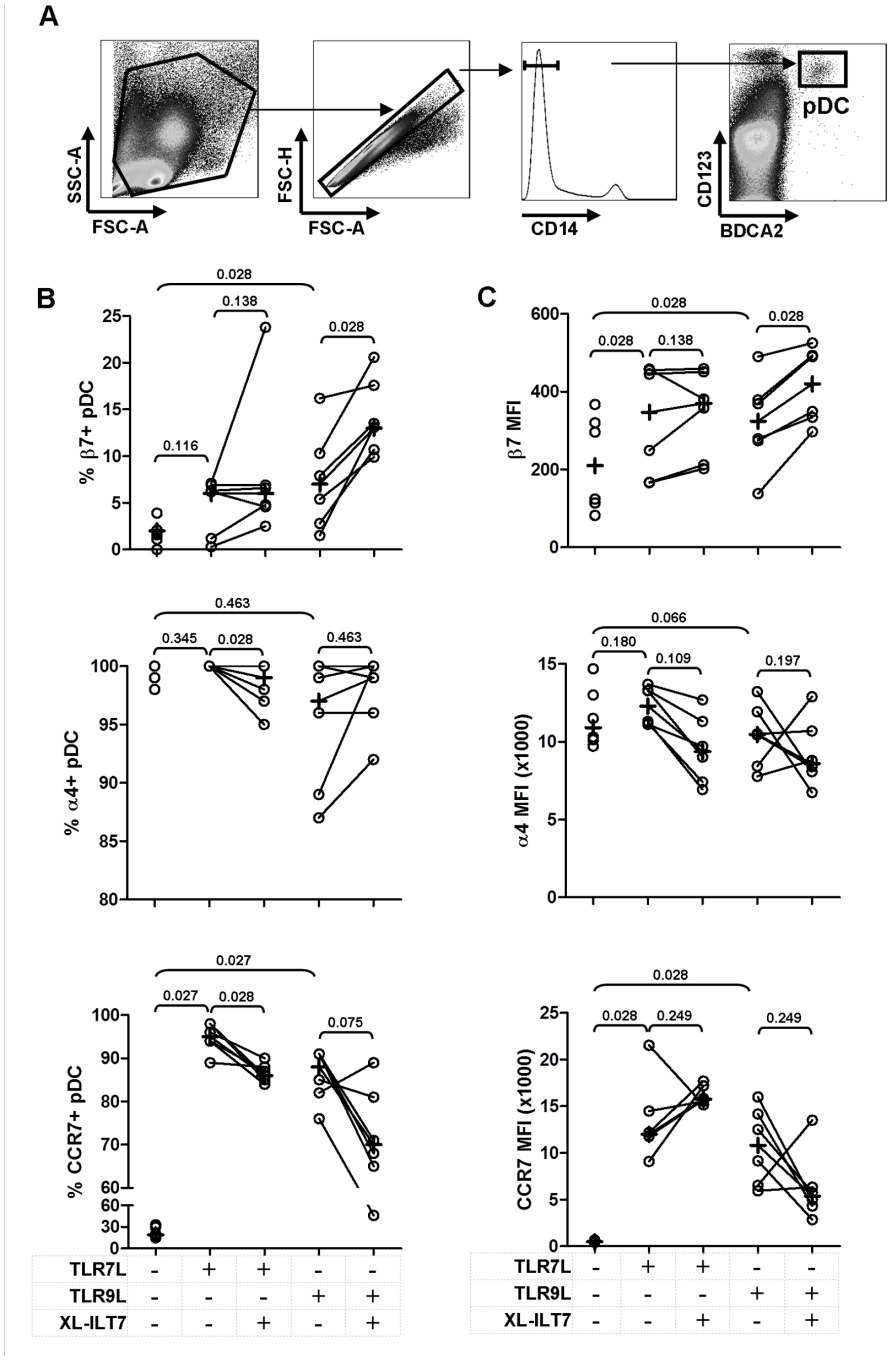
Effect of ILT7 cross-linking on TLR7/9-induced expression of migration markers by pDC. Flow cytometry dot-plots showing the gating strategy used to identify pDC are shown in panel **A**. Frequency of β7+, α4+ and CCR7+ pDC (**B**) and mean fluorescence intensity (MFI) of β7, α4 and CCR7 on pDC (**C**) in PBMC stimulated with TLR7L or TLR9L in the absence (isotype control) or presence of XL-ILT7. In each plot, dots indicate individual experiments and crosses indicate medians for each condition. Numbers above brackets indicate P values for pair wise comparisons (Wilcoxon sign rank test).

ILT7 cross-linking did not significantly affect recovery of pDC (CD14- BDCA2+ CD123+, Fig. 2A) after overnight stimulation with TLR7L (median = 0.037% IQR = 0.016–0.052%, versus median = 0.033% IQR = 0.015–0.053%) or TLR9 (median = 0.033% IQR = 0.015–0.048%, versus median = 0.026% IQR = 0.018–0.039%).

### ILT7 Cross-linking Modulates TLR7/9-induced Expression of Migration Markers by pDC

We analysed pDC (CD14- BDCA2+ CD123+, Fig. 2A) for the expression of α4/β7 and CCR7, which are associated with migration to gut mucosal tissue and secondary lymphoid organs, respectively [22], [23]. Freshly isolated PBMC were cultured overnight in presence or absence of TLR7/9L and pre-incubated or not with plate bound XL-ILT7 Ab. PBMC stimulation with TLR9L induced a significant upregulation in both the frequency of β7+ pDC and β7 MFI, whereas β7 upregulation by TLR7L tested significant only when MFI was analysed (Fig. 2B and 2C). XL-ILT7 significantly enhanced the positive effect of TLR9L on β7 expression (Fig. 2B and 2C). As expected, the frequency of α4+ pDC approached 100% in all conditions tested (Fig. 2B). XL-ILT7 induced a mild, albeit statistically significant decrease in α4+ pDC in presence of TLR7L (Fig. 2B), but no significant effect was observed in response to TLR9L or when α4 expression on a per cell basis, as measured by α4 MFI, was considered (Fig. 2C). Both TLR7L and TLR9L induced a significant upregulation of CCR7 expression in pDC, measured both as frequency of CCR7+ cells and MFI (Fig. 2B and 2C). Interestingly, XL-ILT7 resulted in a mild but statistically significant downregulation of TLR7-induced CCR7+ pDC, and a similar trend was observed in response to TLR9L, albeit not statistically significant (Fig. 2B).

### ILT7 Cross-linking Regulates TLR7/9-induced Expression of Costimulatory Molecules by pDC

**Figure 3:**
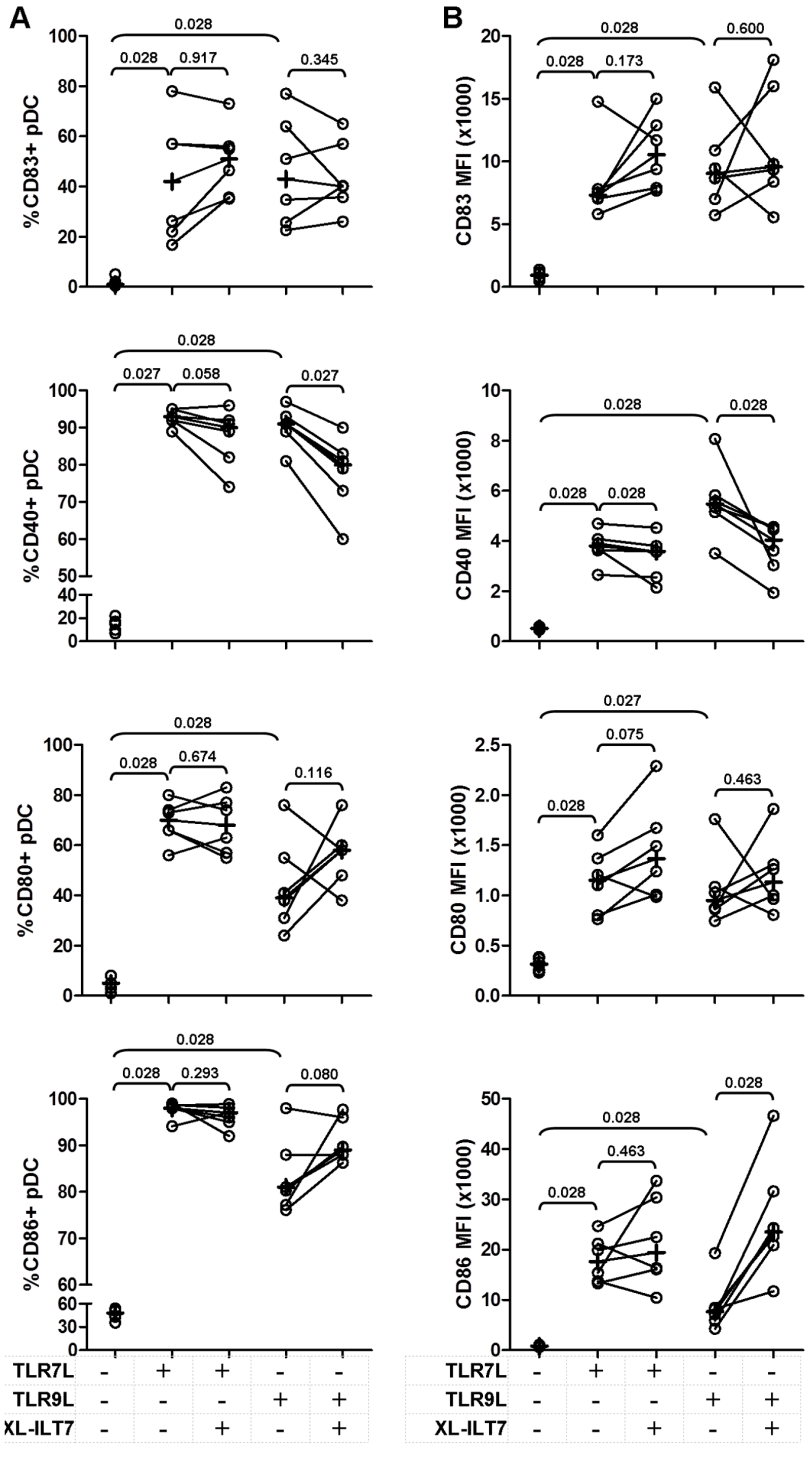
Effect of ILT7 cross-linking on TLR7/9-induced expression of activation and costimulatory markers by pDC. Frequency of CD83+, CD80+, CD40+ and CD86+ pDC (**A**) and MFI of CD83, CD80, CD40 and CD86 on pDC (**B**) in PBMC stimulated with TLR7L or TLR9L in the absence (isotype control) or presence of XL-ILT7. In each plot, dots indicate individual experiments and crosses indicate medians for each condition. Numbers above brackets indicate P values for pair wise comparisons (Wilcoxon sign rank test).

We evaluated the expression of the activation marker CD83 and the costimulatory molecules CD40, CD80 and CD86 by pDC in the same experiments described in Figure 1. As expected, we observed a significant increase in the expression of all markers analyzed in response to TLR7/9L stimulation, compared to untreated cells (Fig. 3A and 3B). TLR7/9L-induced CD83 upregulation was not affected by XL-ILT7 (Fig. 3A and 3B). Similarly, XL-ILT7 did alter TLR7/9-mediated upregulation of CD80 by pDC (Fig. 3A and 3B). Surprisingly, addition of XL-ILT7 to TLR9L induced a significant decrease in the frequency of CD40+ pDC compared to TLR9L stimulation alone (Fig. 3A); a similar trend was observed in response to TLR7L, approaching statistical significance. In addition, XL-ILT7 caused significant decreases in CD40 MFI in both TLR7L- and TLR9L-stimulated cells (Fig. 3B). Conversely, XL-ILT7 further enhanced CD86 MFI in TLR9L- but not TLR7L-stimulated pDC (Fig. 3B).

**Figure 4:**
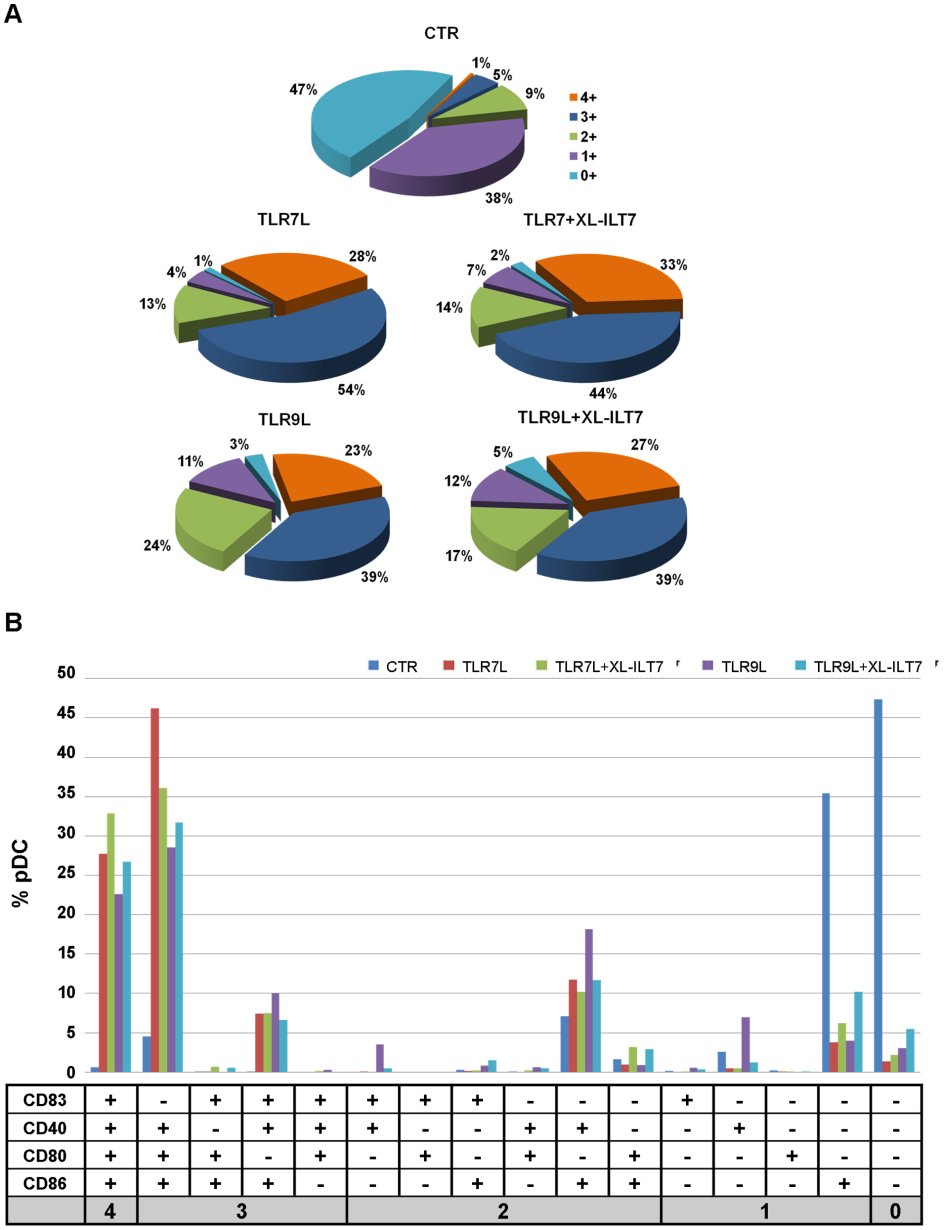
Co-expression of activation and costimulatory molecules on pDC. The data presented in Figure 2 were analyzed for co-expression of multiple markers. **A**) Pie charts showing frequency of pDC expressing 0 (light blue), 1 (violet), 2 (green), 3 (dark blu), or 4 (orange) of the markers analysed (CD83, CD80, CD40 and CD86) in PBMC cultured overnight in media alone (CTR) or stimulated with TLR9L or TLR7L in presence or absence of XL-ILT7 Ab. **B**) Histograms showing the frequency of pDC expressing different combinations of CD83, CD80, CD40 and CD86. In both **A** and **B**, median from experiments performed on N = 6 independent donors are shown.

We evaluated the co-expression of CD83, CD40, CD80 and CD86 by pDC. Only 6% pDC expressed at least three markers of interest in unstimulated pDC (Fig. 4A). Stimulation of PBMC with TLR7L or TLR9L induced co-expression of at least three markers in 82% and 62% pDC, respectively (Fig. 4A). Interestingly, after stimulation with TLR7L or TLR9L in presence of XL-ILT7, co-expression of at least three of the molecules analysed was still observed on 77% and 66% pDC, respectively (Fig. 4A). In all conditions the majority of triple positive pDC co-expressed CD80, CD86 and CD40 (Fig. 4B).

These data collectively suggest that ILT7 cross-linking may exert minor effects on the relative expression of different costimulatory molecules, but does not interfere with pDC maturation into APC, measured as overall expression of costimulatory molecules.

### Effect of ILT7 Cross-linking on pDC Antigen-presentating Activity

**Figure 5:**
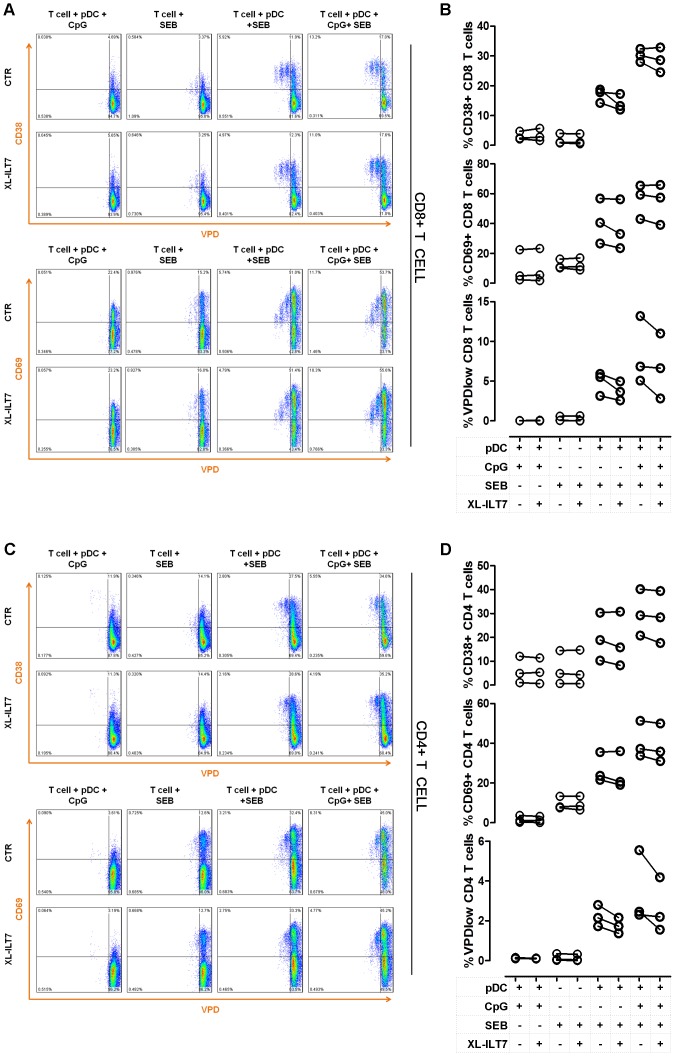
Effect of ILT7 cross-linking on T cell activation in a pDC-T cell co-culture system. Flow cytometry dot plots (**A** and **C**) showing the expression of CD38 and CD69 in relation to violet proliferation dye (VPD) dilution in CD8 T cells (**A**) and CD4 T cells (**C**) co-cultured with autologous pDC (1∶10), in presence of different combinations of TLR9L (CpG), XL-ILT7 and staphyloccoccal enterotoxin B (SEB). One representative example of experiments performed on N = 3 independent donors is shown. Summary of results obtained in N = 3 independent experiments for CD38 expression, CD69 expression and proliferations (%VPDlow) in CD8 (**B**) and CD4 (**D**) T cells in pDC-T cell cocultures in presence of different combinations of TLR9L (CpG), XL-ILT7 and SEB. In **B** and **D**, dots indicate individual experiments.

In order to evaluate the influence of XL-ILT7 on the ability of pDC to stimulate T cell activation and proliferation, we co-cultured purified pDC with autologous T cells in presence of different combinations of TLR9L, XL-ILT7 and the T cell superantigen staphylococcal enterotoxin B (SEB). We analysed the expression of the T cell activation markers CD38 and CD69 in relation to T cell proliferation. As expected, both CD4 and CD8 T cell did not show any proliferative response when cultured alone with SEB or when co-cultured with autologous pDC without SEB, independent of whether TLR9L was present or not (Fig. 5A–D). Conversely, in presence of SEB and pDC, both CD4 and CD8 T cells proliferated and showed upregulation of the activation markers CD38 and CD69. As expected, the expression of the early activation marker CD69 gradually decreased in proliferating cells, as indicated by lower CD69 MFI in late T cell generations (Fig. 5A–D). T cell activation and proliferation in response to SEB was enhanced in T cell-pDC co-cultures stimulated with TLR9L, consistent with pDC maturation into APC (Fig. 5A–D). Increased T cell activation and proliferation in presence of TLR9L was observed also when cells were co-cultured under XL-ILT7 conditions (Fig. 5A–D).

These data suggest that ILT7 cross-linking does not impair the ability of pDC to mature into functional APC in response to TLR stimulation, despite suppressing IFN-α production.

## Discussion

Persistent pDC activation has been shown to lead to harmful consequences for the immune system in both murine models [14], [15] and humans [6], [11]–[13]. When chronically activated, pDC contribute to the detriment of antiviral and antitumor adaptive immunity through persistent IFN-I and IDO overexpression [6], [24]. The signalling pathways leading to pDC differentiation into APC or IFN-I-producing cells (IPC) are mutually exclusive, and pDC which have matured into fully competent APC may not respond to TLR stimulation by producing IFN-I [25], [26]. Thus, regulatory pathways which modulate IFN-I and IDO activity whilst preserving APC function may be of particular interest as candidate immunotherapeutic targets for chronic infections and cancer.

We previously confirmed that ILT7, a surface molecule selectively expressed by human pDC, potently suppress TLR7/9-induced IFN-I [21]. However, we showed that ILT7 expression was rapidly downregulated in pDC which differentiated and matured upon *in vitro* culture [21]. We hypothesised that ILT7 may provide a homeostatic mechanism rather than a negative feedback control on activated pDC [21].

Here we found that XL-ILT7 may modulate the ability of the TLR7/9-stimulated pDC to migrate to the lymph nodes or mucosal tissues. The integrin α4/β7 is involved in the trafficking of different leukocytes, including lymphocytes, NK, DC and macrophages, from the blood to the gut mucosa [22]. In contrast, expression of the chemokine receptor CCR7 by circulating lymphocytes is a fundamental factor for lymph node (LN) entry via high endothelial venules [27], [28]. The expression of CCR7 on human pDC is controversial. Some studies reported high level of CCR7 expression on circulating pDC without, however, conferring responsiveness to CCR7 ligands [29], [30]. Conversely, other studies suggest low expression levels of CCR7 on resting pDC [31]–[33]. However, it is well established that, upon stimulation with TLR ligands, both murine and human pDC increase the expression of CCR7, resembling the mDC activation profile [30], [34], [35]. We observed an increase of TLR-induced expression of β7 integrin, and a parallel decrease CCR7 expression upon XL-ILT7. Of note, ILT7 expression decreases with pDC maturation and activation, which in turn promote CCR7 expression [21]. Thus, ILT7 and CCR7 appear to be mutually exclusive, in that ILT7 cross-linking inhibits CCR7 upregulation and activated CCR7-expressing pDC downregulate ILT7. These data are consistent with the hypothesis we that XL-ILT7 may function as a control mechanism on the activation of immature circulating pDC, by both preventing IFN-α secretion and migration to secondary lymphoid organs, while favouring their retention in the gut mucosa. Extensive *in vivo* experiments may be necessary to determine whether the effect exerted by XL-ILT7 on pDC migration markers is biologically significant.

XL-IL7 did not interfere with the ability of pDC to mature into APC. The analysis of activation and costimulatory markers revealed that, in response to TLR stimulation, XL-ILT7-treated pDC retained the expression of CD83 and CD80 and further increased the expression of CD86. CD40 is normally up-regulated when DC migrate from the peripheral blood to draining lymph nodes as a consequence of microbial challenge [36]. Thus, the observed downregulation of CD40 by XL-ILT7 is consistent with the reduction of CCR7 expression. Importantly, despite the relative changes in CD40 and CD86 expression, the vast majority of pDC showed a phenotype consistent with mature APC following TLR stimulation, independent of XL-ILT7. Although XL-ILT7 caused a mild reduction of CD38 and CD69 expression, and slightly reduced proliferation in CD4 and CD8 T cell subsets in some experiments, it did not prevent TLR9L-induced enhancement of T cell stimulation by pDC in presence of SEB. Thus, XL-ILT7-treated pDC preserved the ability to stimulate T cell activation and proliferation in response to TLR9 stimulation in presence of the superantigen SEB.

Taken together, the data from the present study and our previous report [21] suggest a role for ILT7 in the modulation of immature blood pDC differentiation into either APC or IPC. Thus, in conditions of ILT7 cross-linking, TLR7/9 stimulation may favor the maturation of pDC into APC. Conversely, in the absence of ILT7 cross-linking, TLR7/9 stimulation may lead to pDC differentiation into IPC. Mature ILT7-cross-linked APC-differentiated pDC may partially redistribute between LN and mucosal sites to allow antigen processing and presentation to T cells in the absence of IFN-I- and IDO-dependent cytostatic and cytotoxic mechanisms.
